# Anti-Restriction Protein, KlcA_HS_, Promotes Dissemination of Carbapenem Resistance

**DOI:** 10.3389/fcimb.2017.00150

**Published:** 2017-05-02

**Authors:** Wei Liang, Yingzhou Xie, Wei Xiong, Yu Tang, Gang Li, Xiaofei Jiang, Yuan Lu

**Affiliations:** ^1^Department of Laboratory Medicine, Huashan Hospital, Shanghai Medical College, Fudan UniversityShanghai, China; ^2^State Key Laboratory for Microbial Metabolism and School of Life Sciences and Biotechnology, Shanghai Jiaotong UniversityShanghai, China

**Keywords:** *K. pneumoniae*, *KlcA*_*HS*_, Type I RM systems, horizontal gene transfer, transformation

## Abstract

Carbapenemase-producing *Klebsiella pneumoniae* (KPC) has emerged and spread throughout the world. A retrospective analysis was performed on carbapenem-resistant *K. pneumoniae* isolated at our teaching hospital during the period 2009–2010, when the initial outbreak occurred. To determine the mechanism(s) that underlies the increased infectivity exhibited by KPC, Multilocus Sequence Typing (MLST) was conducted. A series of plasmids was also extracted, sequenced and analyzed. Concurrently, the complete sequences of *bla*_KPC−2_-harboring plasmids deposited in GenBank were summarized and aligned. The *bla*_KPC−2_ and *KlcA*_*HS*_ genes in the carbapenem-resistant *K. pneumoniae* isolates were examined. *E. coli* strains, carrying different Type I Restriction and Modification (RM) systems, were selected to study the interaction between RM systems, anti-RM systems and horizontal gene transfer (HGT). The ST11 clone predominated among 102 carbapenem-resistant *K. pneumoniae* isolates, all harbored the *bla*_KPC−2_ gene; 98% contained the *KlcA*_*HS*_ gene. *KlcA*_*HS*_ was one of the core genes in the backbone region of most *bla*_KPC−2_ carrying plasmids. Type I RM systems in the host bacteria reduced the rate of pHS10842 plasmid transformation by 30- to 40-fold. Presence of the anti-restriction protein, KlcA_HS_, on the other hand, increased transformation efficiency by 3- to 6-fold. These results indicate that RM systems can significantly restrict HGT. In contrast, KlcA_HS_ can disrupt the RM systems and promote HGT by transformation. These findings suggest that the anti-restriction protein, KlcA_HS_, represents a novel mechanism that facilitates the increased transfer of *bla*_KPC-2_ and *KlcA*_*HS*_-carrying plasmids among *K. pneumoniae* strains.

## Introduction

Since carbapenemase-producing *Klebsiella pneumoniae* (KPC) was first reported in the USA in 2001, it has emerged worldwide as a multidrug-resistant hospital pathogen and a significant problem in treating infectious diseases (Yigit et al., 2001). KPC infections often reach mortality rates between 23 and 75% due, in part, to the lack of effective antimicrobial agents (Koraimann and Wagner, 2014). Carbapenem antibiotics are ineffective in treating more than half the patients infected with *K. pneumoniae* due to drug resistance (Pitout et al., 2015). This is particularly problematic because carbapenems are the antibiotics of last resort in treating multidrug-resistant gram-negative bacteria. Carbapenemases in clinical, *K. pneumoniae* isolates are encoded mainly by the *bla*_KPC_ gene, which is carried by many different plasmids (Naas et al., 2008; Nordmann et al., 2011). The horizontal transfer of resistance gene-containing plasmids is a crucial mechanism for disseminating antimicrobial resistance (Carattoli, 2013). The exchange of genetic material by mobile elements, e.g., plasmids and phages, between closely and distantly related bacterial species is influenced by three horizontal gene transfer (HGT) mechanisms: conjugation, transformation, and transduction (Brown-Jaque et al., 2015). Due to these mechanisms, bacteria can achieve significant genetic diversity by acquiring DNA from distantly-related species. This plays a key role in evolution (e.g., acquiring antibiotic resistance), contributing to the fitness and diversity of prokaryotes (Wiedenbeck and Cohan, 2011; San Millan et al., 2015).

Restriction and Modification (RM) systems, which are widespread among prokaryotes (>50% possess genes that encode at least one RM system type), are major barriers to HGT (Veiga and Pinho, 2009; Serfiotis-Mitsa et al., 2010; Vasu et al., 2012; Roberts et al., 2015). Four RM systems (I–IV) are classified dependent upon the complexity of their structure and function (Tock and Dryden, 2005). Type I RM systems, e.g., EcoKI, are composed of the products of three genes: *hsdR* (R, restriction), *hsdM* (M, methylation), and *hsdS* (S, sequence specificity). Type I RM systems can protect host bacteria from foreign DNA by recognizing specific DNA sequences and incising them with restriction endonuclease activity. Notably, Type I RM systems exhibit two seemingly inconsistent enzymatic activities: restriction endonucleases (REase) and methyltransferase (MTase) (Tock and Dryden, 2005). REase recognizes, cleaves and degrades invading foreign DNA, which usually lacks specific modification (e.g., methylation). Type I RM-associated MTase, on the other hand, can convert hemimethylated DNA to the completely methylated form, which resists REase activity. The evidence to date suggests that RM systems are an imperfect barrier to foreign DNA. Plasmids employ a number of strategies to avoid restriction (Wilkins, 2002; Tock and Dryden, 2005).

HGT is directly responsible for the spread of antibiotic resistance genes among pathogenic bacteria derived from both clinical and environment backgrounds (Thomas and Nielsen, 2005; McMahon et al., 2009). An exploration of the mechanism by which mobile genetic elements circumvent barriers such as RM systems during HGT is urgently needed. Multiple anti-RM genes within mobile elements is one likely explanation (Belogurov et al., 2000; McMahon et al., 2009; Serfiotis-Mitsa et al., 2010; Balabanov et al., 2012; Chen et al., 2014). Anti-RM gene products disrupt or negate the RM defense systems and, consequently, promote HGT (Webb et al., 1996; McMahon et al., 2009).

Here, we report that *bla*_KPC−2_ and *KlcA*_*HS*_ genes coexisted in essentially all carbapenem-resistant *K. pneumoniae* isolated at Huashan Hospital. KlcA_HS_ exhibits a high degree of homology with reported anti-restriction proteins KlcA_136_ (43% identity and 66% similarity) and ArdB (30% identity and 58% similarity); both these proteins possess anti-restriction activity and promote transduction (Kamachi et al., 2006). The present study was the first to demonstrate the ability of KlcA_HS_ to facilitate transformation by disrupting RM systems. These findings suggest that KlcA_HS_ is a novel contributor to the spread of carbapenem-resistant *K. pneumoniae*.

## Methods and materials

### Rate of carbapenem resistance

The carbapenem resistance data for *K. pneumoniae* isolated between 2006 and the third quarter of 2016 were obtained using WHONET software, a computerized microbiology laboratory data management and analysis program that, among other things, informs drug-policy decisions and preventive measures (Agarwal et al., 2009). Antimicrobial susceptibility testing was performed using the K-B agar diffusion method. Briefly; all specimens were inoculated onto MHA agar plates and incubated at 35°C for 16–18 h. Ten micrograms imipenem and meropenem disks were used to detect carbapenem-resistant *K. pneumoniae*. Isolates were included in this study if the diameter of the inhibition zone was ≤19 mm. If more than one isolate of the same genus was obtained from a single patient, only one isolate was used. The results were interpreted according to the American Clinical and Laboratory Standards Institute standard (CLSI, 2012).

### Retrospective analyses of MLSTs

One hundred and two carbapenem-resistant *K. pneumoniae* isolates derived from 74 sputum, 20 urine, and 8 blood samples were collected by the microbiology section, Huashan Hospital, during the period 2009–2010, when the initial outbreak at our hospital occurred. In addition to meeting the criteria listed in the preceding paragraph, each isolate was determined to be carbapenem-resistant at imipenem or meropenem concentrations ≥4 μg/mL based upon the 2012 Clinical and Laboratory Standard Institutes standards [imipenem (*S* = ≤1; *I* = 2; *R* = ≥4 μg/mL), and/or meropenem (*S* = ≤1; *I* = 2; *R* = ≥4 μg/mL)]. MLST was performed on all isolates according to the literature using seven conserved housekeeping genes (*gapA, infB, mdh, pgi, phoE, rpoB*, and *tonB*; Diancourt et al., 2005).

### Plasmid extraction, sequencing, and analysis

Five plasmids (pHS062105-3, pHS082416, pHS092753, pHS092839, and pHS10505) were extracted from carbapenem-resistant *K. pneumoniae* isolated at our hospital. pHS062105-3, the first plasmid extracted, was completely sequenced; the others were sequenced partially according to the literature (Shen et al., 2016). A map of pHS062105-3 was generated using the web server (Grant and Stothard, 2008).

### Analysis of complete plasmid gene sequences containing *bla*_KPC−2_ deposited in GenBank

Complete plasmid sequences were searched in GenBank using the restrictive words “*bla*_KPC−2_ and plasmid complete sequence;” repeated records were deleted. All plasmid sequences harboring the *bla*_KPC−2_ and *KlcA*_*HS*_ genes were aligned with the reference sequence, pHS10842, using Blast Ring Image Generator software (Alikhan et al., 2011).

### Detection of *bla*_KPC−2_ and *KlcA*_*HS*_ genes

The *bla*_KPC−2_ and *KlcA*_*HS*_ genes were detected among the carbapenem-resistant *K. pneumoniae* isolates by PCR and primers (P1/P2 and P3/P4, Table 1) designed using Primer Premier 6.22 software (Premier Biosoft, Palo Alto, CA).

**Table 1:** **Primers used in this study**.

**No**	**Primer name**	**Sequence (5′–3′)**	**Product size (bp)**
P1	KPC-2F	TGTAAGTTACCGCGCTGAGG	
P2	KPC-2R	CCAGACGACGGCATAGTCAT	584
P3	KlcA_HS_F	GGCTTATTGGCTTATGTGG	
P4	KlcA_HS_R	GTAGAGGCAAGCGGTAAT	145
P5	Site1F	CCTGGAGTACAGGCGATCGCCTTGCCCTGCTTCTGGAACTGCTG	
P6	Site1R	GGGCAAGGCGATCGCCTGTACTCCAGGATCGGCCAGGCG	13,935
P7	Site2F	CACAGGCGGTCGCGCTGGGTTCCTTCTTCTTGCCGCCAG	
P8	Site2R	GAAGGAACCCAGCGCGACCGCCTGTGCAAGCAGCTCG	13,928
P9	SX1F	TACAGGCGCACCTTGTCGTT	
P10	SX1R	GCGAGGGTGGGCAGATGAC	223
P11	SX2F	GCGGTCGAACACGTGTGTG	
P12	SX2R	TCAATCCAGCCGTCTCGCG	410
P13	CX1F	CGCGCAGTTCCAGCACCTCC	
P14	CX1R	AAGACCTCGGTAAGTTCGAG	420
P15	CX2F	CTTGACGTAATTGCGCAGCGT	
P16	CX2R	TCATCGAGCTCTATGCCGAC	341
P17	KlcA_HS_-F	ACGGTGTCATATGATGCAAACAGAACTTAA	
P18	KlcA_HS_-R	GCTAGAATTCCTAGTCTATTGCGGCCAAG	426
P19	T7primer F	TAATACGACTCACTATAGG	
P20	T7terminator R	GCTAGTTATTGCTCAGCGG	

*The underlined nucleotide sequences shown in P17 and P18 denote the NdeI and EcoRI recognition sites*.

### Strains, plasmids, and antibiotics

*E. coli* strains, selected to assess RM and anti-RM activities *in vivo* (Table 2), were the gift of Dr. D.T. Dryden (University of Edinburgh, UK). Ampicillin (AMP) and kanamycin (Kan) were purchased from Sigma-Aldrich Corp. (St. Louis, MO, USA), and added to the culture media where indicated.

**Table 2:** **Strains and plasmids used in this study**.

**Strain/plasmid**	**Comment**	**Source**
***E. coli***		
DH5α	Cloning strain	Novagen
NM1049	Type IA RM system, EcoKI, Cm^R^	Prof. Dryden
NK354	Type IB RM system, EcoAI	Prof. Dryden
NK402	Type IC RM system, EcoR124I	Prof. Dryden
NM1009	Type ID RM system, StySBLI	Prof. Dryden
NM1261	RM system null, R^−^M^−^	Prof. Dryden
**PLASMID**		
pHS10842	Transformation AMP^R^, KP125892	Our Lab
pET24a	Transformation, Kan^R^	Stratagene

### Preparation of competent cells

A set of *E. coli* strains that carry different Type I RM systems was made competent by a modification of the protocol described by Chan et al. (2013). Briefly, bacteria in LB broth were grown to OD600 ≈ 0.5, collected by centrifugation, washed and resuspended in ice-cold 100 mM CaCl_2_ in 10% (v/v) glycerol. The OD600 of each strain was adjusted to the same value, dispersed into aliquots and stored at −70°C until use.

### Correlate the RM system and transformation efficiency

A series of *E. coli* strains with different RM systems listed in Table 2 were transformed with equivalent moles of pHS10842 plasmid. The transformants of each strain were counted.

### Correlate EcoKI enzyme recognition sites and transformation efficiency

The two EcoKI recognition sites in pHS10842 were knocked out using a modification of the protocol described by Zhang et al. (2014). The P5/P6 and P7/P8 primer pairs were designed based upon the adjacent sequences outside the two extremes of each EcoKI recognition site using Primer Premier 6.22 software (Table 1). The primers were used to amplify the entire pHS10842 sequence except for each EcoKI recognition site. The target DNA fragment was amplified using a high-fidelity PCR Kit (Takara, Dalian, China). The PCR-product was electrophoresed, the target band was recovered and the DNA was purified using a Gel Extraction kit (E.Z.N.A.®Gel Extraction kit, Omega Biotek, Norcross, GA, USA). The linear DNA was cyclized into mutant pHS10842 with the HieffClone One Step Cloning Kit according to the manufacturer's instruction (Yeasen, Shanghai, China).

Competent, RM-deficient cells were transformed with the cloning products generated in the preceding paragraph. EcoKI recognition site-deleted pHS10842 clones were selected by colony-PCR with the P9/P10 and P11/P12 primer pairs (Table 1), inoculated into LB broth containing 100 μg/ml AMP, and incubated overnight. Each site deleted pHS10842 was obtained, confirmed using DNA sequencing technology with P13/P14 or P15/P16 primer pairs, and named Δsite1 and Δsite2 pHS10842, respectively. Δsite1 plus Δsite2 pHS10842 was obtained using Δsite2 pHS10842 as the DNA template and the same methodology. The same number of *E. coli* competent cells (NM1049, Type IA RM system [EcoKI]-positive) was transformed with equal moles of Δsite1, Δsite2, and Δsite1 plus Δsite2 pHS10842 under identical experiment conditions, and the transformants were quantified.

### Construct recombinant pET24a-KlcA_HS_

The *KlcA*_*HS*_ gene was PCR-amplified with primers P17/P18 (Table 1) using pHS092839 extracted from a clinical, carbapenem-resistant *K. pneumoniae* isolate as the DNA template. The primers P17/P18 were predesigned with NdeI and EcoRI recognition sites. The PCR-products were combined with pET24a vector, digested with NdeI and EcoRI (New England Biolabs, Inc., Beijing, China), purified and ligated with T4 DNA ligase (New England Biolabs); competent *E. coli* DH5α cells were subsequently transformed. Recombinant pET24a-KlcA_*HS*_-expressing clones were identified using colony PCR and confirmed by DNA sequencing both strands with primers P19/P20 (Table 1) to assure no mistakes were introduced during amplification.

### Compare the transformation efficiencies of pET24a and recombinant pET24a-KlcA_*HS*_

To evaluate the anti-restriction activity of KlcA_HS_, competent, RM system-defective cells were transformed with unmethylated wild-type pET24a or recombinant pET24a-KlcA_HS_ plasmids, and the transformation efficiencies were compared. The two unmethylated plasmids were extracted with Plasmid Mini Kit I (E.Z.N.A.® Plasmid Mini Kit I), each was quantified using NANODROP (NanoDrop2000, Thermo Fisher Scientific, USA), and the molecular weights were calculated with Snap gene viewer software (GSL Biotech, available at snapgene.com). Equal moles of pET24a and recombinant pET24a-KlcA_HS_ were added to competent cells, mixed and placed on ice before heat shocking at 42°C for 90 s. Afterwards, the cells were placed on ice, then added to LB broth and allowed to recover at 37°C with shaking. Aliquots were transferred to LB agar plates containing 25 μg/ml Kan and incubated overnight. The transformant colonies were counted the next day. Transformation efficiency ratios were calculated by dividing the pET24a-KlcA_HS_ transformants by the pET24a transformants.

The anti-modification function of KlcA_HS_ was estimated by comparing the transformation efficiencies of wild pET24a and recombinant pET24a-KlcA_HS_ extracted from the transformants obtained in the step above. Each transformant was transferred to fresh LB broth and cultured overnight; both plasmids were extracted from strains carrying different Type I RM systems using E.Z.N.A.® Plasmid Mini Kit I. Competent cells were transformed with the newly extracted plasmids, pET24a-KlcA_HS_ and pET24a. Subsequently, the transformants of both pET24a-KlcA_HS_ and pET24a were counted. Transformation efficiencies ratios were calculated by dividing the pET24a-KlcA_HS_ transformants with the pET24a transformants. Both the anti-restriction and anti-modification activities of KlcA_HS_ were considered significant *in vivo* at ratios >2 (McMahon et al., 2009).

### Statistical analysis

The results were analyzed using the SPSS 13.0 software program compatible with Windows. Data derived from more than two groups were compared by one-way analysis of variance (ANOVA) followed by a Dunnett's or Tukey's test to identify the groups that differed significantly (*P* < 0.05).

## Results

### Carbapenem-resistance rates of *K. pneumoniae* isolated between 2006 and 2016

**Figure 1:**
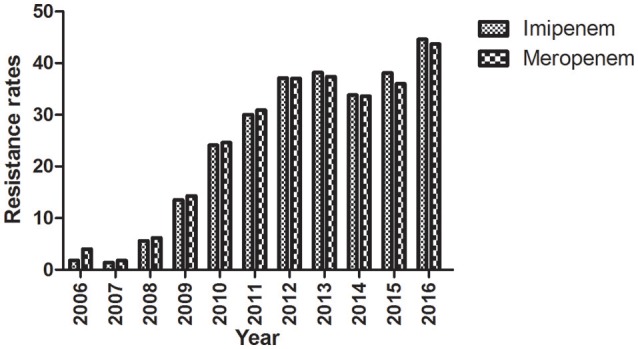
**Imipenem- and meropenem-resistance rates from 2006 to 2016**. Clinical *K. pneumoniae* isolates were obtained during the period 2006–2016 and the resistance to imipenem and meropenem was determined.

The first carbapenem-resistant *K. pneumoniae* isolate was identified at Huashan Hospital in 2006. The years 2007 and 2008 represented a temporary transition period. The outbreak of carbapenem-resistant *K. pneumoniae* occurred in 2009; the rate has increased yearly thereafter suggesting the trend will continue (Figure 1).

### MLSTs of carbapenem-resistant *K. pneumoniae* isolates

The MLSTs of 102 clinical, carbapenems-resistant *K. pneumoniae* isolates obtained between 2009 and 2010 were determined in an effort to define their relationship (Diancourt et al., 2005). All isolates belonged to a predominant epidemic clone, ST11, which exhibits a distinctive allelic profile based upon seven housekeeping genes that differentiates these isolates from those that belong to other epidemiologic clusters.

### Analyses of the plasmid sequences derived from the carbapenem-resistant *K. pneumoniae* isolates

**Figure 2:**
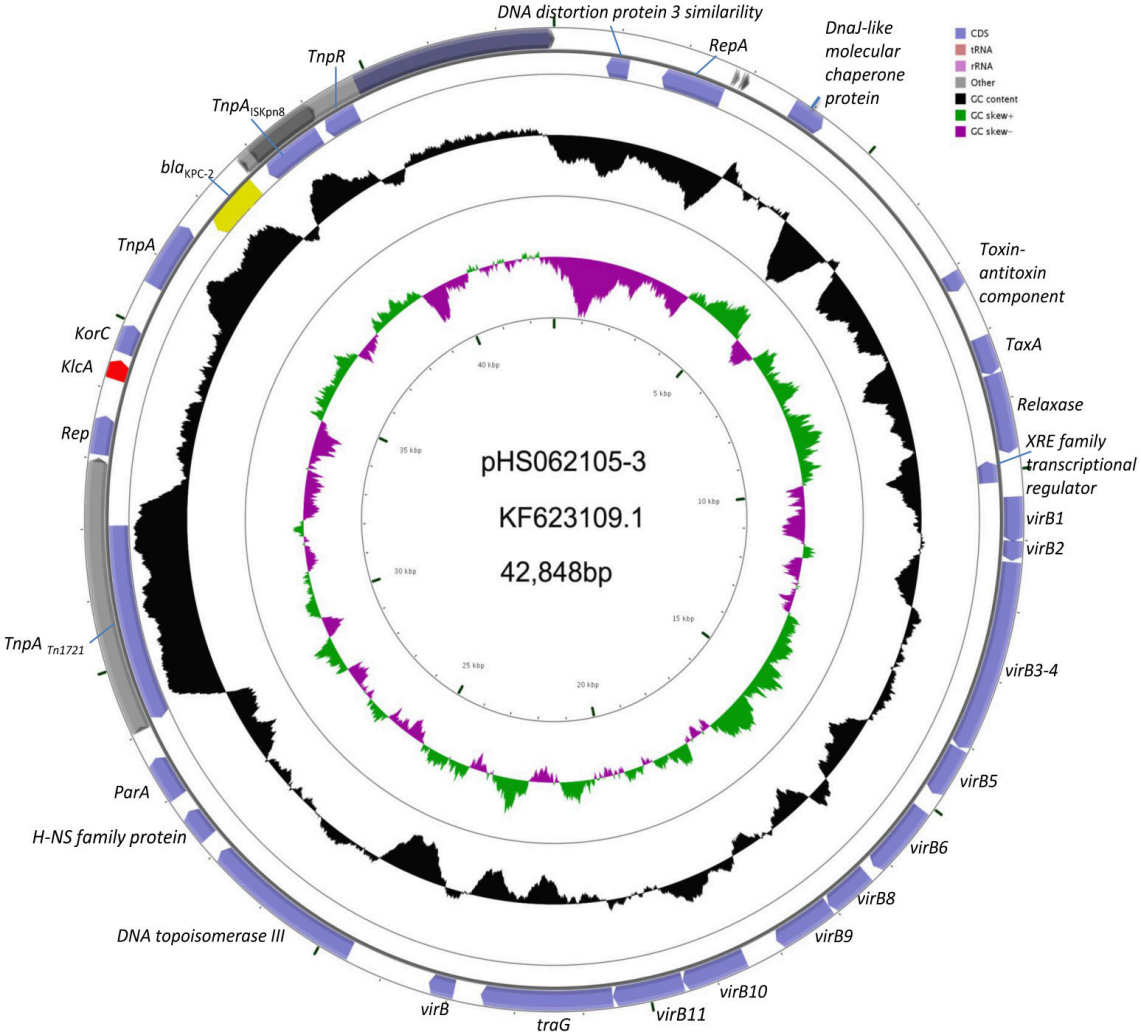
**Schematic map of pHS062105-3**. The *KlcA*_*HS*_ and *bla*_KPC−2_ genes are demarcated by red and yellow, respectively, in the schematic map of pHS062105-3 shown.

Experiments were undertaken to explore the connection between *KlcA*_*HS*_ and *bla*_KPC−2_. The complete plasmid sequence of pHS062105-3 and the partial sequences of pHS082416, pHS092753, pHS092839, and pHS10505 were obtained by sequencing technology. These sequences were deposited in the GenBank under accession number NC_023331.1, KF724507.1, KF826293.1, KF724506.1, and KF826292.1, respectively. The results showed that *KlcA*_*HS*_ and *bla*_KPC−2_ genes coexisted in the plasmids analyzed (Figure 2).

### Analysis of the genetic structure of *bla*_KPC−2_-harboring plasmids

**Figure 3:**
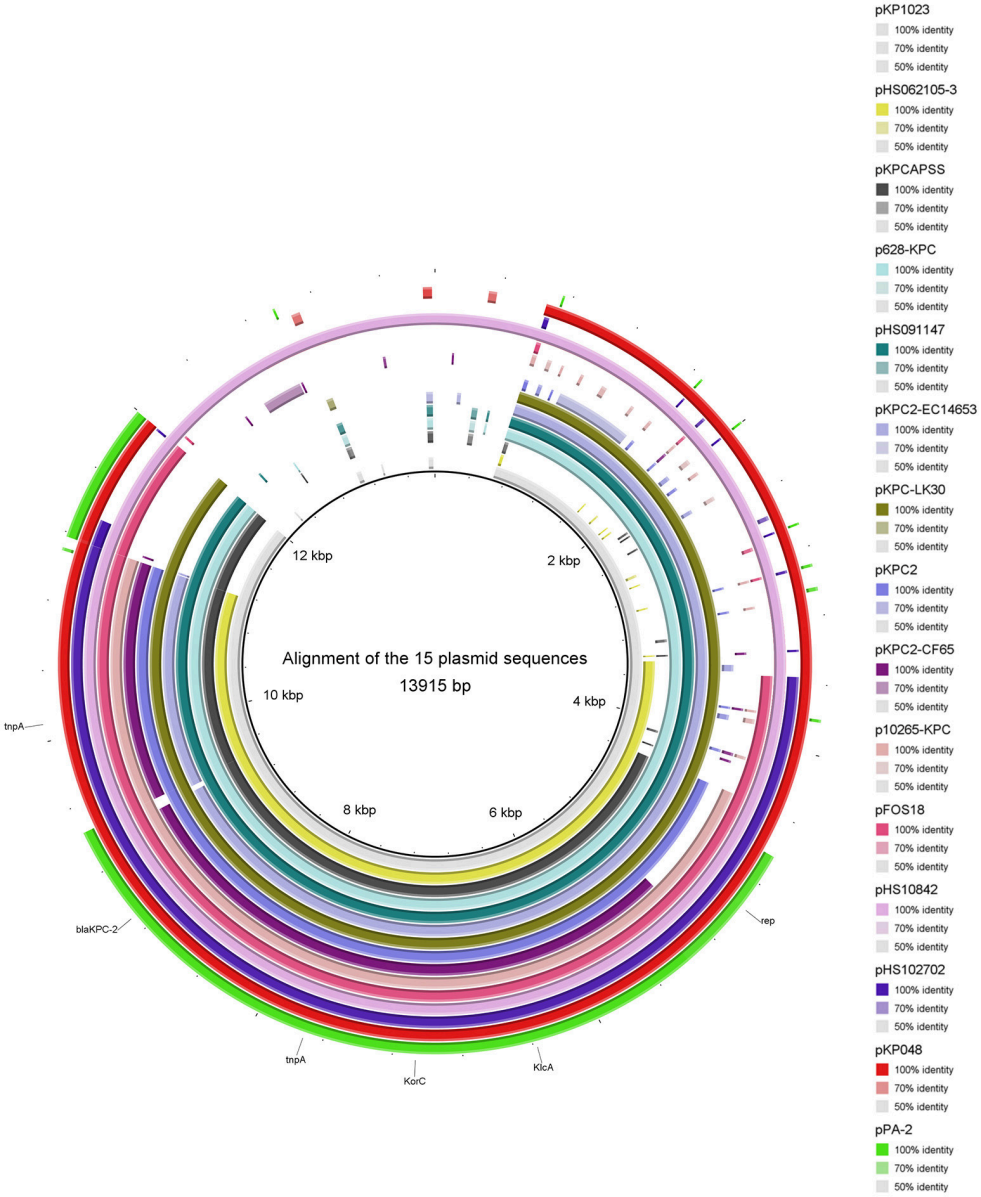
**Alignment of 15 plasmid sequences that harbor ***KlcA***_***HS***_**. The backbone region of plasmids that harbor *bla*_KPC−2_ was confirmed. The *KlcA*_*HS*_ gene combined with other genes constitutes the backbone region of many plasmids submitted mainly from China.

Thirty-one complete plasmid sequences were analyzed and summarized (Table 3), showing that the common genetic environment near the *bla*_KPC−2_ gene was categorized arbitrarily into three groups. The first group had a Tn*1721*-like genetic background, which is often encountered in China. Within this group, 15 plasmid sequences harbored a backbone region in which the *KlcA*_*HS*_ and *bla*_KPC−2_ genes coexisted (Figure 3). The second group had a Tn*4401*-like genetic structure. The third group was comprised of plasmids that had no obvious common feature.

**Table 3:** **Thirty-one completely sequenced ***bla***_**KPC−2**_-harboring plasmids searched in GenBank**.

**Accession**	**Plasmid**	**Region**	**Length (bp)**	**KlcA^a^_HS_**	**Bacterium**	**KPC element**
KC609322	pPA-2	France	7,995	Y	*P. aeruginosa*	Tn*1721*
KP125892	pHS10842	China	13,915	Y	*E. coli*	Tn*1721*
KJ653815	pFOS18	China	23,939	Y	*K. pneumoniae*	Tn*1721*
NC_023907	pHS102707	China	69,453	Y	*E. coli*	Tn*1721*
KU578314	p10265-KPC	China	38,939	Y	*P. aeruginosa*	Tn*1721*
KU176944	pKPC2_CF65	China	40,947	Y	*C. freundii*	Tn*1721*
NC_023331	pHS062105-3	China	42,848	Y	*K. pneumoniae*	Tn*1721*
KR014106	pKPC2	China	44,451	Y	*A. hydrophila*	Tn*1721*
KX236178	pHSO91147	China	121,348	Y	*K. pneumoniae*	Tn*1721*
KP008371	PKPCAPSS	Russia	127,970	Y	*K. pneumoniae*	Tn*1721*
KP893385	KP1034	China	136,848	Y	*K. pneumoniae*	Tn*1721*
FJ628167	pKP048	China	151,188	Y	*K. pneumoniae*	Tn*1721*
KC405622	pKPC-LK30	Taiwan	86,518	Y	*K. pneumoniae*	Tn*1721*
KP868646	pKPC2-EC14653	China	88,214	Y	*E. cloacae*	Tn*1721*
KP987218	p628-KPC	China	105,008	Y	*K. pneumoniae*	Tn*1721*
JX283456	pKPN101-IT	Italy	107,748	N	*K. pneumoniae*	Tn*4401*
KF874499	pGR-3913	Greece	113,640	N	*K. pneumoniae*	Tn*4401*
KF874498	pGR-1870	Greece	116,047	N	*K. pneumoniae*	Tn*4401*
KC609323	pCOL-1	France	31,529	N	*P. aeruginosa*	Tn*4401*
JX461340	pKpS90	France	53,286	N	*K. pneumoniae*	Tn*4401*
CP004366	pKPC_FCF13/05	Brazil	53,081	N	*K. pneumoniae*	Tn*4401*
NC_021660	pKPC_FCF/3SP	Brazil	54,605	N	*K. pneumoniae*	Tn*4401*
JX397875	pKP1433	Greece	55417	N	*K. pneumoniae*	Tn*4401*
KU665642	pG12-KPC-2	Norway	111,926	N	*K. pneumoniae*	Other
NC_024967	pYD626E	USA	72,800	N	*E. coli*	Other
KC958437	pKo6	China	65,549	N	*K. pneumoniae*	Other
KP125893	HS08204	China	133,031	N	*K. pneumoniae*	Other
KR091915	pKPC-DK05	Korea	56,728	N	*K. pneumoniae*	Other
KC788405	pKPC-LKEc	Taiwan	145,401	N	*K. pneumoniae*	Other
LN610760	pCF8698_KPC2	Germany	54,036	N	*C. freundii*	Other
JX430448	pBK32179	USA	165,295	N	*K. pneumoniae*	Other

a *Y and N indicate the presence or absence of a KlcA_HS_ gene in the corresponding plasmid*.

### Detection of the *bla*_**KPC−2**_ and *KlcA*_*HS*_ genes in carbapenem-resistant *K. pneumoniae*

The results of PCR-based screening demonstrated the presence of the *bla*_KPC−2_ gene in all 102 clinical, carbapenem-resistant *K. pneumoniae* isolates. The *KlcA*_*HS*_ gene was found in 98% (100/102) of those isolates. These findings document the common coexistence of *bla*_KPC−2_ and *KlcA*_*HS*_ gene in carbapenem-resistant *K. pneumoniae* isolates, at least those recovered at Huashan hospital.

### RM systems of the host restrict transformation

**Figure 4:**
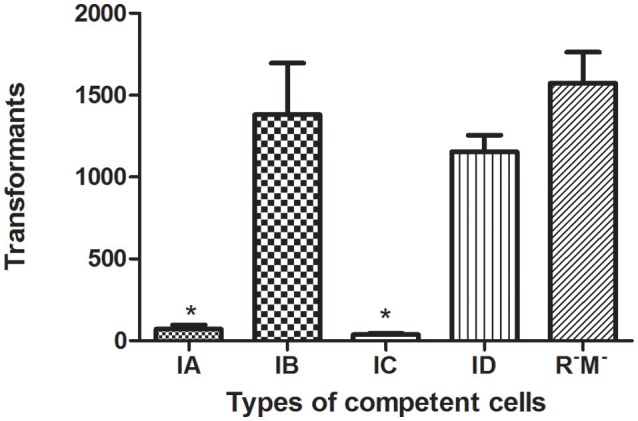
**Transformation of ***E. coli*** strains that possess different RM systems**. The X axis indicates the absence (R^−^M^−^) and presence of a Type I RM system, represented by restriction enzymes EcoKI (IA), EcoIA (IB), EcoR124I (IC), and StySBLI (ID), expressed by an *E. coli* strain. The Y axis denotes the number of unmethylated pHS10842 transformants. Data are the means ± SD derived from three similar experiments. ^*^Significantly less than R^−^M^−^; *P* < 0.05.

Bacterial hosts possess RM systems, which cleave internalized plasmids that possess restriction enzyme sites recognized by RM system-associated REases. As a consequence, the rate of transformation is reduced. Bacterial strains that carry different RM systems exhibited varied rates of transformation by an equivalent number of pHS10842. *E. coli* strains that possess EcoKI (Type IA, 5′-AACNNNNNNGTGC-3′) and EcoR124I (Type IC, 5′-GAANNNNNNRTCG-3′) REases can recognize and degrade internalized pHS10842. On the other hand, internalized pHS10842 lacks sequences recognized by EcoAI (Type IB, 5′-GAGNNNNNNNGTCA -3′) and StySBLI (Type ID, 5′-GGTANNNNNNTCG-3′) REases and, therefore, remains intact. The transformation of *E. coli* strains that possessed Type IA or IC system enzymes (i.e., EcoKI and EcoR124I, respectively) were reduced significantly relative to transformation of strains that possessed IB, ID, and null RM systems (Figure 4).

### Restriction enzyme recognition sites exert a critical effect on transformation

**Figure 5:**
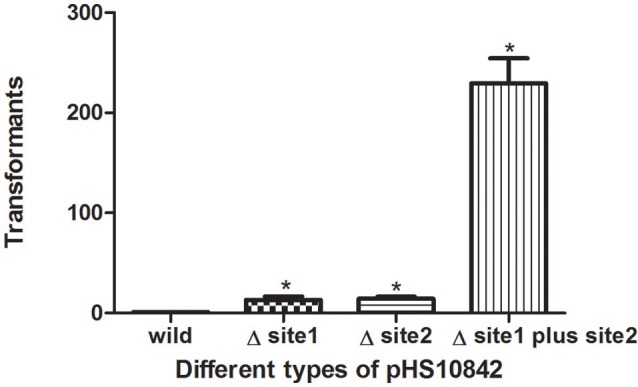
**Restriction enzyme recognition site and transformation efficacy**. *E. coli* strain (NM1049, EcoKI^+^) transformed with pHS10842 that lacks one or both restriction enzyme recognition sites. The number of transformants is indicated. Data are the means ± SD derived from three similar experiments. ^*^Significantly greater than wild-type; *P* < 0.05.

The presence of restriction enzyme recognition sites in plasmids exert a negative impact on transformation. Compared to wild-type pHS10842, the number of transformants was increased when either of the two EcoKI recognition sites was deleted. When both EcoKI recognition sites were disrupted, the rate of transformation increased dramatically (Figure 5).

### *KlcA*_*HS*_ expression promotes transformation efficiency

**Figure 6:**
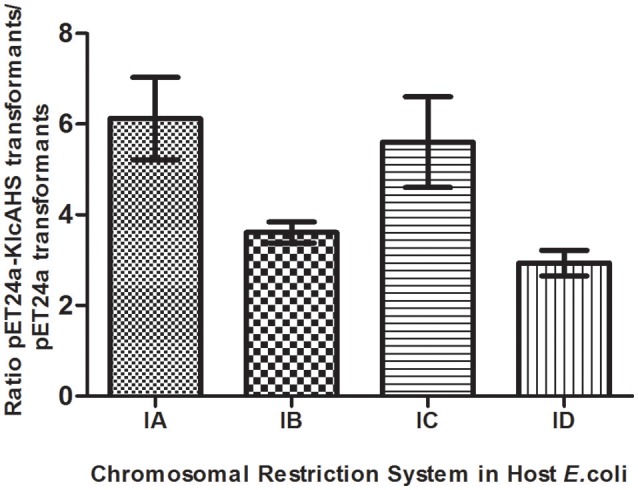
**Anti-restriction activity of KlcA_**HS**_**. Four Type I RM systems, represented by restriction enzymes EcoKI (IA), EcoIA (IB), EcoR124I (IC), and StySBLI (ID), are indicated on the X-axis. All plasmids used were extracted from an R^−^M^−^ strain. The assay was repeated in triplicate. Data are the means ± SD.

Experiments were initiated to determine the ability of KlcA_HS_ to counteract the negative effect(s) of the host RM system on transformation. KlcA_HS_ promoted the transformation efficiency of pET24a by 3- to 6-fold in *E. coli* strains that carry IA-D RM systems. The anti-restriction activity varied when subjected to different Type I RM system enzymes (Figure 6). In contrast, the anti-modification activity expressed by KlcA_HS_ was not affected significantly; the efficiency of transformation varied ≤2-fold (data not shown).

## Discussion

The rate of carbapenem resistance among *K. pneumoniae* isolated at Huashan Hospital rose steadily during the past decade. Currently, the rate of imipenem resistance is 44.6% compared to 1.8% in 2006. The initial outbreak of carbapenem–resistant *K. pneumoniae* occurred between 2009 and 2010. Carbapenem resistance is increasing worldwide (Nordmann et al., 2011; Hu et al., 2016; Tian et al., 2016). The increase in carbapenem-resistant *K. pneumoniae* resulted in the availability of fewer antibiotics, and a huge economic and social burdens. Little is known about the exact mechanism of carbapenemase dissemination. In this study, we found that Type I RM systems and the anti-restriction protein, KlcA_*HS*_ exerted major effects on HGT. Disparate rates of transformation occurred when *E. coli* strains with different RM systems were transformed with equivalent concentrations of pHS10842. No specific sequences in pHS10842 are recognized by EcoAI (Type IB) and StySBLI (Type ID) REases, but *E. coli* strains that possess EcoKI (Type IA) and EcoR124I (Type IC) REases can recognize and degrade pHS10842. Consequently, the transformation rate of *E. coli* strains that possess restriction enzymes EcoKI and EcoR124I was 30- to 40-fold less than strains that possess EcoAI, StySBLI, or no RM system, indicating the significant ability of RM systems to restrict HGT. The rate of transformation increased ~100-fold when both enzyme recognition sites in pHS10842 were deleted and *E. coli* strains that possessed a Type IA RM system (i.e., EcoKI) were transformed with the resultant plasmid. When either of the two recognition sites was deleted, the transformation rate increased ~10-fold; no significant difference existed between the two sites. Clearly, the plasmid sequence and the RM system carried by the host were crucial factors affecting HGT. The distribution of RM systems among *K. pneumoniae* isolates is sporadic and not well-studied; most systems were predicted using bioinformatic tools (Roberts et al., 2015). As such, model *E. coli* strains were selected to examine KlcA_*HS*._ activity.

Anti-restriction systems are important counterstrategies employed by mobile genetic elements to evade the RM systems in recipient host bacteria. RM systems have been studied extensively as primitive defense mechanisms (Tock and Dryden, 2005; Vasu et al., 2012; Roberts et al., 2015; Roer et al., 2015; Oliveira et al., 2016). RM systems comprise a major, imperfect barrier to HGT; anti-restriction proteins, such as ArdA, ArdB, ArdC, ArdD, and KlcA_136_, possess anti-restriction activity in transduction (Belogurov et al., 2000; McMahon et al., 2009; Serfiotis-Mitsa et al., 2010; Balabanov et al., 2012). Those anti-restriction proteins augmented the plating efficiency of lambda phage more than 10-fold (Serfiotis-Mitsa et al., 2010). Our understanding of anti-RM systems is inadequate, however, especially regarding their role in disseminating drug-resistance genes among clinical bacterial isolates. Here, anti-RM gene *KlcA*_*HS*_ and *bla*_KPC−2_ coexisted in the backbone structure in many complete plasmid sequences submitted mainly by Chinese institutes. Furthermore, all the carbapenem-resistant *K. pneumoniae* isolates collected at our hospital at the beginning of the outbreak harbored the *bla*_KPC−2_ gene; 98% of them contained the *KlcA*_*HS*_ gene. The biological significance of this finding remains obscure, however; whether it contributes to HGT is presently unclear. Transformation was selected as a means of HGT to explore the role KlcA_*HS*_.

Transformation involves the uptake and functional establishment of DNA by recipient bacteria. Many pathogenic bacteria, e.g., *Staphylococcus* and *Streptococcus*, are naturally transformable (Lorenz and Wackernagel, 1994). Indeed, ~1% of bacterial species are naturally transformable, facilitating their ability to obtain beneficial characteristics from distantly-related species (Jonas et al., 2001). The low-level expression of *KlcA*_*HS*_ by unmethylated wild pET24a, which normally occurs in the absence of induction (Nie et al., 2013), increased the rates of transformation in the present study by 6.1-, 3.6-, 5.6-, and 2.9-fold when the recombinant plasmid was transformed into a series of *E. coli* strains that harbored IA, IB, IC, and ID RM systems, respectively. A single target sequence in pET24a is recognized by the Type I RM enzyme, EcoKI (IA), or EcoR124I (IC); however, no target sequence is recognized by restriction enzyme EcoAI (IB) or StySBLI (ID). Consequently, the enhanced rate of transformation as a function of *KlcA*_*HS*_ expression was greater for the Type IA and IC strains than for the Type IB and ID strains. Intriguingly, *KlcA*_HS_ expression also increased the rate of transformation in *E. coli* strains that possessed Type IB and ID RM systems. Presumably, in the latter cases, KlcA_*HS*_ increased the mobility of plasmids by unknown strategies in addition to disrupting the RM system. Furthermore, when both wild pET24a and recombinant pET24a-KlcA_HS_ were extracted from the transformants of each *E. coli* strain, then transformed back into the same competent strain, the transformation rates of the two plasmids never varied more than 2-fold. It is conceivable that the extracted plasmids were modified by RM enzymes, rendering them immune to restriction. It seems reasonable to conjecture that KlcA_HS_ possesses anti-restriction, but no anti-modification, function which is consistent with the findings of other investigators (Serfiotis-Mitsa et al., 2010). It is probable, therefore, that KlcA_HS_ anti-restriction protein facilitates HGT in the natural microbial community by transformation, especially among certain bacterial species.

Transformation is a means of HGT that facilitate the movement of foreign DNA into new bacterial strains. The balance between the RM systems in the recipient and the anti-restriction systems in the mobile genetic elements constitute one of most important factors affecting the rate of HGT. The mechanism(s) by which KlcA_*HS*_ affects the RM systems is elusive. The effect of KlcA_HS_ on other HGT mechanisms, i.e., conjugation and transduction, is unclear and the subject of considerable, ongoing effort in our laboratory.

In conclusion, KlcA_HS_ was found effective in negating the Type I RM systems, probably facilitating HGT by functioning as an anti-restriction protein. Conceivably, the frequent occurrence of the *KlcA*_*HS*_ gene in carbapenem-resistant *K. pneumoniae* isolates contributes to the yearly increase in these organisms isolated in our hospital.

## Author contributions

XJ and YL conceived and designed the study. WL performed the experiments and wrote the paper. YX, WX, YT, and GL provided suggestions and helped perform the experiments. All authors have read and approved this manuscript.

## Funding

This study was supported by grants from the National Natural Science Foundation of China (NSFC 81571365, 81372141).

### Conflict of interest statement

The authors declare that the research was conducted in the absence of any commercial or financial relationships that could be construed as a potential conflict of interest.
